# Cocaine and amphetamine-regulated transcript prepropeptide gene (CARTPT) polymorphism interacts with Diet Quality Index-International (DQI-I) and Healthy Eating Index (HEI) to affect hypothalamic hormones and cardio-metabolic risk factors among obese individuals

**DOI:** 10.1186/s12967-020-02208-z

**Published:** 2020-01-09

**Authors:** Mahsa Mahmoudi-Nezhad, Mahdieh Abbasalizad Farhangi, Houman Kahroba

**Affiliations:** 1 Nutrition Research Center, Tabriz University of Medical Sciences, Tabriz, Iran; 2 Drug Applied Research Center, Tabriz University of Medical Sciences, Attar-Neishabouri Ave, Golgasht St, Tabriz, 5165665931 Iran; 3 Molecular Medicine Research Center, Tabriz University of Medical Sciences, Tabriz, Iran

**Keywords:** Diet quality, Obesity, Gene-diet interaction, CARTPT, Metabolic risk factors, AgRP, α-MSH

## Abstract

**Background:**

Epidemiologic studies show that cocaine- and amphetamine-regulated transcript prepropeptide (CARTPT) gene polymorphism modifies diet-obesity relationships. However, the interaction between CARTPT gene polymorphism and diet quality indices have not been investigated yet. The current study was aimed to evaluate the interaction between major dietary indices including Diet Quality Index-International (DQI-I) and Healthy Eating Index (HEI)-2015 and CARTPT gene rs2239670 variants among apparently healthy obese Iranians.

**Methods:**

This cross-sectional study was carried out by employing 288 apparently healthy obese adults aged 20–50 years with a BMI of 30–40 kg/m^2^. Diet quality was evaluated by Diet Quality Index-International (DQI-I) and Healthy Eating Index-2015 (HEI-2015) using a 132-items semi-quantitative validated food frequency questionnaire. The CARTPT gene rs2239670 polymorphism was genotyped by polymerase chain reaction-restriction fragment length polymorphism (PCR–RFLP) technique. Blood concentrations of glycemic markers, lipid profile, α-melanocyte stimulating hormone (MSH) and agouti-related peptide (AgRP) were also measured. ANCOVA multivariate interaction model was used to analyze gene-diet interactions.

**Results:**

The significant interactions were identified between CARTPT gene polymorphism and HEI, affecting BMR (P_Interaction_ = 0.003), serum glucose (P_Interaction_ = 0.009) and high density lipoprotein cholesterol HDL concentrations (P_Interaction_ = 0.03) after adjusting for the effects of sex and age. Also we found gene-diet interaction between CARTPT genotypes and DQI-I in terms of fat mass (FM; P_Interaction_ = 0.02), waist circumference (WC; P_Interaction_ < 0.001), body mass index (BMI; P_Interaction_ < 0.001), basal metabolic rate (BMR, P_Interaction_ < 0.001), serum fasting glucose (P_Interaction_ < 0.01) and AgRP (P_Interaction_ = 0.05) in individuals even after adjusting for potential confounders.

**Conclusion:**

Current study showed the effects of interaction between CARTPT genotype with adherence to HEI and DQI-I scores on obesity-related anthropometric and metabolic risk-factors.

## Introduction

**Fig. 1:**
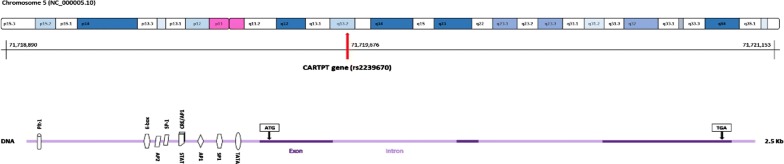
Genomic organization of the CARTPT rs2239670 polymorphism region

Obesity, as a major pandemic health problem, refers to the excessive and abnormal fat accumulation according to the definition of World Health Organization (WHO) [1], resulting from an imbalance between energy intake and expenditure [2]. It is mostly defined as having body mass index (BMI) greater than 30 kg/m^2^ [3]. The prevalence of obesity has been increased dramatically in the past decades [4]. It has been estimated that approximately 1.35 billion and 573 million individuals will be obese and overweight by 2030 [5]. Recent studies show that obesity prevalence have been raised in Iranian adults in last years [5]. The interaction between genetic and life style behaviors including diet and physical activity have been recently attracted much attention in treatment and management of obesity and related disorders. Genetic and molecular epidemiological evidences suggest that there is a clear difference in the susceptibility to obesity and obesity-related disorders according to their genetic traits [6]. Accordingly, the adverse effects of obesity on cardio-metabolic risk factors has strong dependence to genetic variants and therefore, it is necessary to develop a tri-polar approach of gene-nutrient-metabolic risk factor interactions in molecular studies. So, nutrigenetics and nutrigenomics studies can provide an understanding of the intra and inter population phenotypic differences in the effect of obesity on metabolic risk factors [7]. Over the past decades, genome-wide association studies (GWAS) have identified the cocaine- and amphetamine-regulated transcript prepropeptide gene encoding cocaine- and amphetamine-regulated transcript (CART) protein as a neurotransmitter and hormone [8–10], located in the arcuate nucleus of the hypothalamus [11] and implicated in several physiological processes such as neurological disease, anxiety, stress, addiction, depression, endocrine function, specially feeding behavior, weight management and energy homeostasis [10–12]. CART prepropeptide gene located in chromosome 5q13-14 in coexistence of proopiomelanocortin (POMC) neurons [12], contains one exon and two introns [11] and produces short peptide fragments [10, 13] (Fig. 1). Alpha melanocyte-stimulating hormone (α-MSH) as a derivative of POMC along with CART leads to energy expenditure enhancing food intake and appetite inhibition [10]. It has been identified that CART prepropeptide gene polymorphism is associated with obesity [14]. Also Rigoli et al. [15] declared that CART gene single nucleotide polymorphism (SNP) may affect CART expression which is associated with obesity in children. Other study by del-Giudice et al. [16] suggested the possible role of Leu34Phe mutation of CART gene might play an important role in obese phenotype by altering the CART mediated leptin effect on thermogenesis and energy expenditure. Likewise, understanding of gene-diet interactions may be more effective in prevention of the obesity-related phenotypes and their better management. The role of diet as one of lifestyle factors in preventing obesity and relevant non-communicable diseases and also maintaining good health is indisputable [17]. Since food and food ingredients including micro or macro-nutrients are usually consumed in combination of each other in a usual diet and as a consequent they will have numerous synergistic or even inhibitory interactions, it is logical to study their simultaneous effects in the diet rather than studying their isolate actions; dietary pattern approach, accounting complexity of the diet, provides the opportunity to approve the real image of a diet on health outcome [18]. Among priori-defined dietary pattern approaches, as known as ‘diet quality indices’, including diet quality index International (DQI-I) and healthy eating index (HEI) [19, 20] have been known as being cardio-protective. DQI-I is developed to clarify dietary variations among population; whereas, HEI-2015, the most recently updated version is a nutrient density-based approach [21]. In the current study, we aimed to evaluate the interaction between CARTPT genotype and dietary indices and the effects of these interactions on anthropometric and metabolic risk factors in obese individuals.

## Method and materials

### Study population and protocol

The current cross sectional study was conducted in Tabriz, Iran between December 2017 and April 2019. Using convenience random sampling procedure, 288 healthy obese individuals who met the inclusion criterias recruited to the study. Eligible individuals aged 20–50 years old with a BMI of 30–40 kg/m^2^ without prior history of alcohol or drug abuse, any presence of acute or chronic inflammatory disease, hypertension, hepatic, renal or cardiovascular disorders, diabetes mellitus, malignancies, thyroid diseases, and acute or chronic infections. Subjects with a history of weight change more than 5 kg in the last 6 months, use of any medications affecting weight, pregnancy and lactation were excluded from the study. Written informed consent was obtained from all the participants at the beginning of the study. The study protocol was approved and registered by the ethics committee of Tabriz University of Medical Sciences (Ethics number: IR.TBZMED.REC.1397.666).

### Anthropometric assessments

Demographic data including: age, sex, socioeconomic status (SES), marital status, smoking and medical history were completed for each individual in a structured questionnaire by interviewing. Anthropometric assessments including weight, height, waist circumference (WC), and hip circumference (HC) were performed by a trained person. Weight was measured with a Seca scale to the nearest 100 g removing his/her shoes with a lightweight clothing. Height was measured to the nearest 0.5 cm using a local stadiometer in a normal standing position. WC was measured at the narrowest area of waist using a non-stretchable tape measure with a 0.1 cm precision and without any pressure to the body. HC was also measured around the most prominent part of the hip to the nearest 0.1 cm. Waist-to-hip ratio (WHR) was calculated by dividing WC to HC. Systolic and diastolic blood pressure (SBP and DBP) were assessed twice in a relaxing position after 15 min-resting and the mean values were recorded. Body composition was measured through body composition analyzer BC-418-Tanita (United Kingdom). Physical activity level was also evaluated using the international physical activity questionnaire (IPAQ).

### Laboratory assessments

Intravenous blood was collected from all the subjects after 12 h fasting and centrifuged 10 min at 3000 rpm, 4 °C, aliquoted into 1 mL tubes and stored at − 80 °C until assay. Fasting serum glucose, triglyceride (TG), total cholesterol and high-density lipoprotein cholesterol (HDL-C) were measured using a commercial kit (Pars Azmoon, Tehran, Iran). Serum low-density lipoprotein cholesterol (LDL-C) was calculated according to the Friedewald method [22]. Plasma α-MSH and AgRP levels were measured using commercially available enzyme-linked immunosorbent assay (ELISA) kits (Bioassay Technology Laboratory, Shanghai Korean Biotech, Shanghai City, China) according to the manufacturer’s instructions. The minimum detectable doses of α-MSH and AgRP levels were 5.07 ng/L and 1.03 pg/mL, respectively. Serum insulin level was determined by similar ELIZA kit (Bioassay Technology Laboratory, Shanghai Korean Biotech, Shanghai City, China). Homeostasis model assessment-insulin resistance index (HOMA-IR) and quantitative insulin sensitivity check index (QUICKI) were computed according to the following equations [23]:

$${\text{HOMA-UR }} = \, \left[ {{\text{fasting glucose }}\left( {{\text{mmol}}/{\text{L}}} \right) \, \times {\text{ fasting insulin }}\left( {\upmu{\text{IU}}/{\text{mL}}} \right)} \right] \, / \, 22.5$$

$${\text{QUICKI }} = \, 1 \, / \, \left[ {\log {\text{ fasting glucose }}\left( {{\text{mmol}}/{\text{L}}} \right) \, + \, \log {\text{ fasting insulin }}\left( {\upmu{\text{IU}}/{\text{mL}}} \right)} \right]$$

### Dietary assessments

Dietary intake was evaluated by using 132-items semi-quantitative food frequency questionnaire (FFQ) a reliable and validated questionnaire to estimate the food consumption and dietary nutrient intake over the past year [24]. Questionnaires were completed by a well-trained nutritionist, and participants reported the intake frequency and quantity of each food item over the past year. Then, portion sizes of reported foods were converted to gram due to household measurements. Ultimately daily energy and nutrient intake were analyzed using Iranian food composition table (FCT) [25].

### Healthy eating index (HEI)-2015

The Healthy Eating Index (HEI) is a method for diet quality assessment according to dietary guidelines for Americans (DGA) recommendations [21]. It has been structured to examine diet quality and health outcomes associations. HEI-2015 is composed of 13 components, 9 adequacy and 4 moderation components with a total score of 100 points. The highest and lowest consumption of three adequacy components (e.g., whole grains, dairy and fatty acids) scored 10 and 0, respectively. Other Six adequacy components include total fruits (fruit, fruit juice and canned fruit), whole fruits (fruits except fruit juice), total vegetables, seafood and plant proteins, greens and beans, and total protein foods; each scored at 5 for the highest and 0 for the lowest consumption. A maximum of 10 points was given to the lowest consumption of four moderation components includes refined grains, sodium, added sugars, and saturated fats. However, the highest consumption of these items was scored as 0. Intermediate intake of every component was scored proportionally. Higher overall HEI-2015 scores shows greater alignment with DGA recommendation and better diet quality [21].

### DQI-I

The Diet Quality Index—International (DQI-I) is a measure determining overall health effects of diet and provides appropriate nutrition intake recommendations. It takes into account both undernutrition and overnutrition statues. DQI-I could evaluate diet quality in terms of nutritional variety, adequacy, moderation and balance together. Dietary variety scores such as diversity in food groups and protein sources gets 20 points. Adequacy scores contain adequate fruits, vegetables, grains intake obtain 40 points. Moderation scores includes total fat, saturated fat, cholesterol, sodium wile empty calorie foods gets 30 points and lastly overall balance score which consider macronutrient balance and fatty acid ratios receives 10 points. Scores of each components in four categories will be summed to obtain the final DQI score. A higher DQI-I score is considered as a high quality diet indicator [26].

### Genotyping

Blood samples were collected from all individuals in EDTA-containing tubes. Genomic DNA was extracted from blood sample and its concentration was measured using Nano Drop 2000C spectrophotometer. The CARTPT rs2239670 SNP (major allele: G; minor allele: A) was genotyped by polymerase chain reaction-restriction fragment length polymorphism (PCR–RFLP) technique. The forward and backward primers were 5′-CCTGCTGCTGATGCTACCTCT-3′ and 5′-GCGCTTCGATCTGCAACACAC-3′, respectively. PCR was run as follows: denaturation at 94 °C for 30 s, annealing at 60 °C for 30 s, and extension at 72 °C for 20 s, for 35 cycles. The reaction cycles were preceded by a single cycle of denaturation at 94 °C for 5 min and ended with a single cycle of extension at 72 °C for 10 min. The RFLP analysis for the rs2239670 variant was performed by incubating the PCR products with ApaI (Takara, Japan) enzyme. All products were electrophoresed on 3% agarose gels. Fragments containing three possible genotypes were then distinguished: uncut homozygous AA (552 bp), cut heterozygous AG (212, 340 and 552 bp) and cut homozygous GG (340 and 212 bp).

### Statistical analysis

Data were analyzed using SPSS version 22.0. Descriptive measures such as coefficients of skewness and kurtosis, mean and standard deviation (SD) were used to check distribution of data. We used the mean ± SD for reporting normally distributed continuous variables, the median (25th and 75th percentile) for the continuous variables that were not normally distributed, and the frequency (%) for categorical data. General characteristics of subjects across CARTPT genotypes were determined using one-way analysis of variance (ANOVA) and Chi square tests. Multivariate multinomial logistic regression was performed to check out diet quality indices and rs14477313 genotype associations. ANCOVA multivariate model with adjustment to confounders using the general linear model procedure have been used to investigate CART polymorphism (rs2239670) and diet quality indices interactions. P values less than 0.05 were considered statistically significant.

## Results

**Fig. 2:**
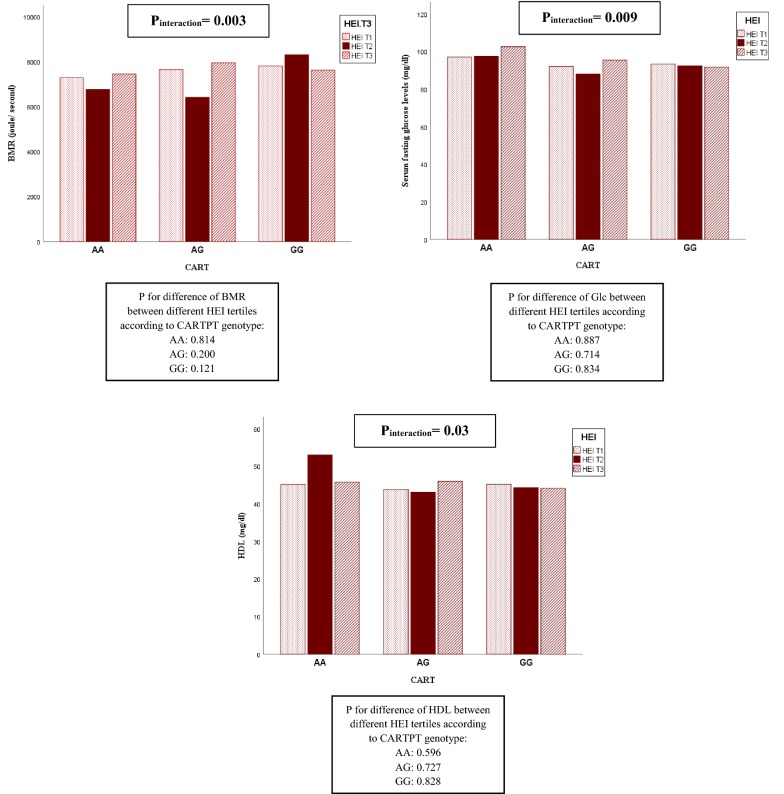
P-for differences between different HEI tertiles according to CARTPT genotype

**Fig. 3:**
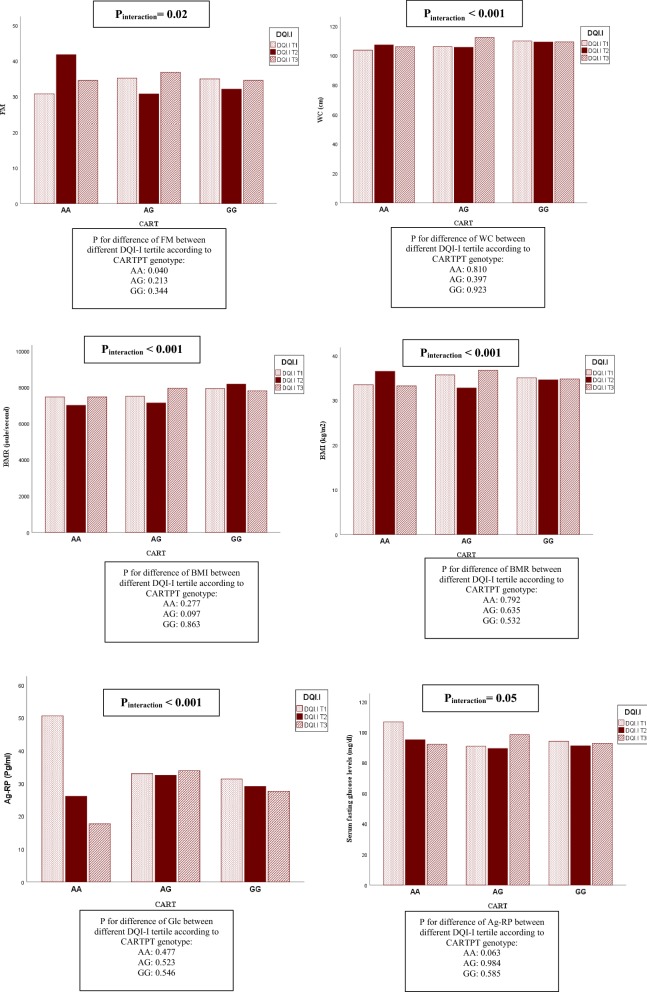
P for differences between different DQI-I tertile according to CARTPT genotype

A total of 288 participants were involved in this cross sectional study with sex distribution of 51.1% and 48.9% in men and women respectively and the allele frequency of A (20.79%), G (79.21%). Basic characteristics of study participants across the CART rs2239670 genotypes have been presented in Table 1. Genotype prevalence among study participants was as follow: AA (5.4%), AG (20.4%), GG (74.1%) and AA (15.6%), AG (20%), GG (64.4%) in men and women respectively. Significant differences had been shown about age distribution among genotypes in men (P < 0.05). Table 2 indicates biochemical parameters of participants according to CARTPT gene rs2239670 polymorphisms. There were statistically significant difference in terms of α-MSH (P = 0.010), AgRP (P = 0.019) in women and glucose (P = 0.05) and HOMA (P = 0.035) in men across CARTPT gene rs2239670 genotypes. While higher amounts of these parameters were observed in AA genotype compared with other genotypes. Figure 2 presents CARTPT-HEI interactions and their effects on several anthropometric and biochemical variables. Accordingly, CARTPT-HEI interactions affect basal metabolic rate (BMR; P_Interaction_ = 0.003), glucose (P_Interaction_ = 0.009), HDL (P_Interaction_ = 0.03) after adjusting for sex and age. The risk of obesity and metabolic syndrome was not homogenous in CART genotype groups, across tertiles of HEI. The lowest BMR was observed in the AG genotype with moderate compliance with the HEI; highest BMR values was observed in highest HEI tertile in AA and AG genotypes. AA genotype had also the highest fasting serum glucose concentrations even in highest adherence to HEI. While being in AG or GG genotype reduced serum glucose concentrations (P = 0.009). Also the lowest HDL was observed in the AG genotype with moderate adherence to the HEI; while its concentrations reduced in highest tertile. As a conclusion, HEI could not modify the adverse effects of being in AA genotype of CARTPT. Whereas, gene-diet interactions were more pronounced for CARTPT DQI-I compared with CARTPT-HEI as represented for FM (P_Interaction_ = 0.02), WC (P_Interaction _< 0.001), BMI (P_Interaction _< 0.001), BMR (P_Interaction _< 0.001), serum fasting glucose (P _Interaction _< 0.001) and AgRP (P_Interaction _= 0.05) even after adjusting for potential confounders (Fig. 3). Higher adherence to DQI-I scores reduced fasting serum glucose and Ag-RP levels in AA genotype; while individuals with lower adherence to DQI-I exhibited a higher serum fasting glucose and Ag-RP concentrations. Accordingly, highest adherence to DQI-I index reduced FM, BMI, fasting serum glucose and Ag-RP in AA genotype while increased BMR values further highlighting the beneficial effects of high adherence to DQI-I to reduce the adverse obesity-related risk factors. The interaction between HEI or DQI-I and other anthropomeric or biochemical parameters were not statistically significant while the related information are provided in supporting information (Additional file 1: Figures S1 and S2).

**Table 1:** Comparison of clinical and laboratory parameters of participants according to CART rs2239670 genotypes

	Women (n = 140)				Men (n = 147)			
	GG (n = 90)	AG (n = 28)	AA (n = 22)	P*	GG (n = 109)	AG (n = 30)	AA (n = 8)	P*
Age (year)	38.41 (7.99)	37.44 (9.06)	35.85 (7.89)	0.567	37.24 (6.42)	42.27 (7.24)	40.40 (5.36)	*0.015*
BMI (kg/m^2^)	36.03 (4.46)	34.89 (3.63)	34.78 (4.22)	0.450	33.63 (2.53)	35.09 (5.52)	33.14 (2.38)	0.231
WC (cm)	105.85 (10.08)	100.34 (8.91)	103.00 (10.71)	0.112	112.63 (6.33)	115.30 (11.95)	111.60 (3.13)	0.390
WHR	0.88 (0.06)	0.86 (0.04)	0.85 (0.05)	0.087	0.99 (0.03)	0.98 (0.04)	1.00 (0.03)	0.588
FM (%)	39.53 (9.10)	36.07 (6.00)	38.24 (7.56)	0.309	28.71 (6.41)	32.18 (10.44)	25.80 (1.60)	0.116
BMR (kcal)	1605.77 (142.95)	1523.15 (134.36)	1605.29 (209.01)	0.130	2222.62 (360.05)	2152.03 (225.11)	2151.81 (146.68)	0.678
PA (min/week)	1477.05 (286.83)	1300.55 (446.64)	1225.07 (368.56)	0.891	3112.16 (522.32)	1164.11 (349.13)	1875.80 (976.37)	0.139
SBP (mmHg)	114.06 (14.82)	114.27 (15.64)	113.21 (18.57)	0.979	117.53 (12.87)	114.27 (28.43)	107.00 (8.36)	0.356
DBP (mmHg)	76.18 (11.84)	79.16 (10.03)	75.07 (13.11)	0.559	76.30 (10.08)	74.11 (19.46)	67.00 (9.08)	0.254

Italic values indicate significance of P value (P < 0.05)

*BMI* body mass index, *WC* waist circumference, *WHR* waist-to-hip ratio, *FM* fat mass, *BMR* basal metabolic rate, *PA* physical activity, *SBP* systolic blood pressure, *DBP* diastolic blood pressure; values for all variables are presented based on mead (SD)

^*^P values based on One-Way ANOVA

**Table 2:** Comparison of clinical and laboratory parameters of participants according to CART rs2239670 genotypes

	Women (n = 140)				Men (n = 147)			
	GG (n = 90)	AG (n = 28)	AA (n = 22)	P*	GG (n = 109)	AG (n = 30)	AA (n = 8)	P*
LDL-C (mg/dL)	116.66 (34.00)	124.32 (37.89)	122.82 (28.47)	0.645	122.07 (29.43)	114.48 (24.87)	125.68 (27.63)	0.563
HDL (mg/dL)	46.40 (9.37)	50.05 (7.87)	47.50 (10.25)	0.347	43.00 (6.89)	39.22 (6.87)	43.00 (6.74)	0.122
Cholesterol (mg/dL)	185.71 (36.83)	191.83 (36.94)	190.50 (33.59)	0.786	191.16 (32.40)	180.05 (27.49)	199.80 (32.87)	0.316
TG (mg/dL)	113.26 (43.56)	87.27 (34.73)	100.85 (34.30)	0.06	130.49 (70.65)	131.72 (62.35)	155.60 (53.83)	0.732
Glucose (mg/dL)	91.08 (10.34)	88.38 (12.08)	95.00 (11.02)	0.235	93.95 (12.82)	96.94 (25.40)	113.80 (39.96)	*0.05*
Insulin (U/mL)	18.36 (9.25)	11.81 (6.77)	15.73 (10.43)	0.329	14.16 (7.63)	13.81 (6.76)	21.16 (11.06)	0.139
HOMA-IR	4.22 (2.18)	2.66 (1.66)	3.68 (2.57)	0.531	3.22 (1.77)	3.38 (1.99)	5.50 (2.52)	*0.035*
QUICKI	0.31 (0.02)	0.34 (0.03)	0.32 (0.03)	0.116	0.32 (0.02)	0.32 (0.02)	0.30 (0.02)	0.143
α-MSH (ng/L)	168.14 (97.74)	274.41 (213.43)	271.55 (231.49)	*0.010*	226.61 (172.86)	201.94 (122.25)	299.61 (249.29)	0.533
AgRP (pg/mL)	24.12 (9.52)	33.94 (21.69)	34.86 (27.11)	*0.019*	33.93 (19.92)	31.5 (13.04)	38.80 (29.97)	0.789

Italic values indicate significance of P value (P < 0.05)

*TC* total cholesterol, *TG* triglyceride, *HDL*-*C* high density lipoprotein cholesterol, *LDL*-*C* low density lipoprotein cholesterol, *HOMA*-*IR* homeostatic model assessment for insulin resistance, *QUICKI* quantitative insulin sensitivity check index, *α*-*MSH* alpha-melanocyte-stimulating hormone, *AgRP*, agouti-related peptide; all variables data are presented based on mean (SD)

^*^P values based on ANCOVA after adjustment for age

## Discussion

To the best of our knowledge, this is the first study investigated the interactions between CARTPT rs2239670 genes with dietary indices in terms of metabolic parameters. We demonstrated that AA genotype was the possible risk factor of metabolic disorders and adherence to higher diet quality index of DQI-I reduced the metabolic risk in obese individuals. While these interactive role was not favorite for HEI. As elucidated above AA homozygote genotype can increase metabolic abnormalities like serum fasting glucose level despite high adherence to HEI and DQI-I and also can increase FM, BMI, AgRP even in high compliance with DQI-I. The role of CARTPT gene in obesity has been studied in several previous works; Two variants, 1457delA and A1475G, in the coding region of CARTPT gene were detected in Danish Caucasians with early onset obesity in Echwald et al. study [27]. In addition, according to the results of Challis study, the A1475G and 1457delA variants are not associated with obesity in Caucasian population in the U.K, and the A1475G SNP was significantly associated with lower WHR, fasting plasma insulin, and fasting triglycerides [28]. Likewise, no association was reported between C1442G variant of CARTPT gene and obesity in a group of obese Pima Indians [29]. In contrast, Yamada et al. [30] demonstrated that the A-156G variant of the CARTPT gene was associated with obesity in the Japanese population. Accordingly, our results were in line with other Asian populations in Korea [31] and Malaysia [32] where the majority of the participants had GG genotype about the genotype prevalence and allele frequency of this gene polymorphism. Moreover, consistent with our findings, there was no significant difference in term of anthropometric measurements and blood pressures among the genotypes [12]. Although the underlying mechanisms for these different results remain poorly understood, differences in dietary habits and other socio-demographic factors in studied populations may contribute to these inconsistent outcomes. Although, no study is available evaluating the CARTPT gene-interactions with dietary indices and their effects on metabolic parameters in obesity, but numerous previous studies are available investigating the interaction between single nucleotide gene polymorphisms with diet or dietary ingredients in the pathogenesis of obesity-related comorbidities; for example the interaction between Mediterranean dietary pattern and FTO gene variants [33], dietary inflammatory index and VEGF genotype [34], and the role of dietary approach to stop hypertension interaction with insulin receptor substrate-1gene expression [35] affecting obesity and obesity related metabolic phenotypes are available in literature. Our findings revealed the interactive role of DQI-I in reducing obesity related metabolic profile including BMI, FM and FSG and AgRP in AA genotype of CARTPT; no human study is available evaluating the relationship between AgRP and CART gene polymorphisms. However, increased AgRP in obesity had been reported in several previous reports [36–38]. The possible underlying mechanism of increased AgRP in obesity is hypothalamic re-sistance to its function due to increased leptin concentrations in obese individuals [39]. In terms of CARTPT-HEI interactions, being in highest tertiels of HEI could not show favorable effects in reducing metabolic risk factors; for example FM increased while HDL decreased in highest adherence to HEI in AA genotype. Similarly, Sanjeevi et al. and Kirwan et al. reported that a whole grain-rich diet with high adherence to HEI score significantly reduced serum HDL concentrations in overweight and obese adults [40, 41].

To date, there is no study regarding the interaction between the CARTPT polymorphisms and dietary indices in relation to cardio-metabolic traits and hypothalamic hormones in obesity and this is the main strength of current study. Our report, partially, emphasize to the beneficial role of higher adherence to DQI-I in reducing the obesity-related risk factors. There are several limitations in this study that should be taken into account. First, the cross-sectional design of the study makes it impossible to infer causality; therefore, more studies with interventional designs are warranted. Second, under-reporting of dietary intakes in obese adults, could be a possible source of report and recall bias affecting the results [42], although the FFQ used in the current study is a validated questionnaire and the information are reliable; moreover, FFQ covers a wide range of dietary ingredients and has more accuracy compared with 24-h recall method in reflection of usual dietary intake in a short period of time; it has been confirmed that FFQ could be more helpful in evaluating the diet-disease relationships [43].

## Conclusion

Our findings revealed an association between the rs2239670 SNP of the CARTPT gene and obesity and exhibited evidences several interactions between this variant and dietary indices in terms of BMR, WC, FM, serum glucose and AgRP particularly in terms of CARTPT–DQI-I associations.

## Supplementary information

**Additional file 1.** The interaction between HEI (Figure S1) or DQI-I (Figure S2) with anthropometric and biochemical variables according to CARTPT genotype.

## Data Availability

All of the data are available with reasonable request from the corresponding author
