# Supplementary material for: Cocaine and amphetamine-regulated transcript prepropeptide gene (CARTPT) polymorphism interacts with Diet Quality Index-International (DQI-I) and Healthy Eating Index (HEI) to affect hypothalamic hormones and cardio-metabolic risk factors among obese individuals
Source: J Transl Med. 2020 Jan 9;18:16. doi: 10.1186/s12967-020-02208-z (PMC6953221; doi:10.1186/s12967-020-02208-z)
Supplement: Supplementary file 1 — Additional file 1. The interaction between HEI (Figure S1) or DQI-I (Figure S2) with anthropometric and biochemical variables according to CARTPT genotype. [file 12967_2020_2208_MOESM1_ESM.docx]

**Title: Cocaine and Amphetamine-Regulated Transcript Prepropeptide gene (CARTPT) polymorphism interacts with Diet Quality Index-International (DQI-I) and Healthy Eating Index (HEI) to affect hypothalamic hormones and cardio-metabolic risk factors among obese individuals**

**Authors: Mahsa Mahmoudi-Nezhad ^1^, Mahdieh Abbasalizad Farhangi ^2^*, Houman Kahroba** **^3^**

^1^ Nutrition Research Center, Tabriz University of Medical Sciences, Tabriz, Iran.

^2^ Drug Applied Research Center, Tabriz University of Medical Sciences, Tabriz, Iran.

^3^ Molecular Medicine Research Center, Tabriz University of Medical Sciences, Tabriz, Iran

**Running title:** CART gene-diet interactions in obesity.

**Corresponding author E-mail**: [abbasalizad_m@yahoo.com](mailto:abbasalizad_m@yahoo.com). Postal code: 5165665931

Attar-Neishabouri Ave, Golgasht St, Tabriz, Iran.

**Additional file**


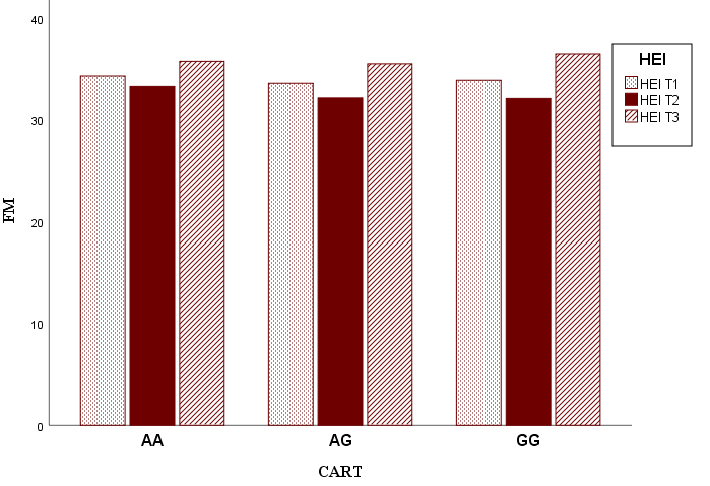

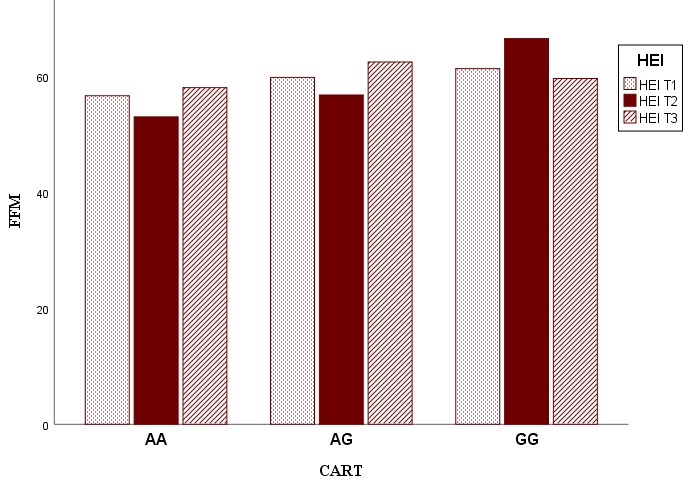

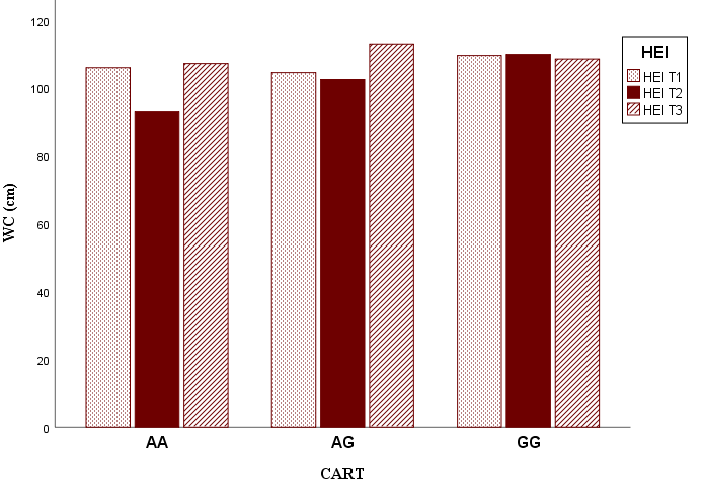

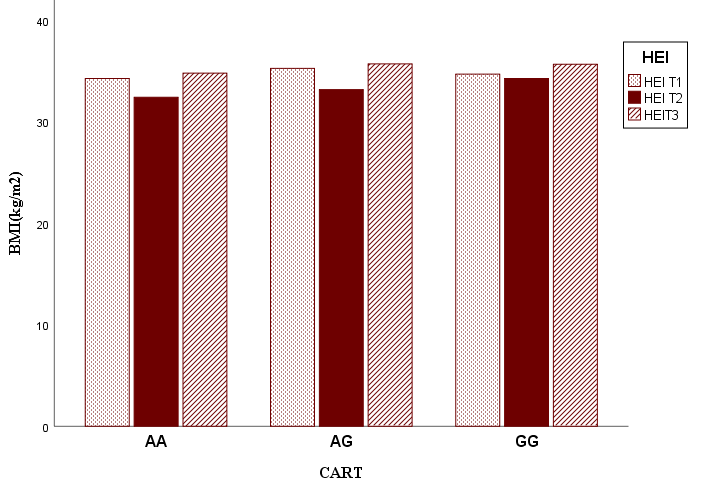

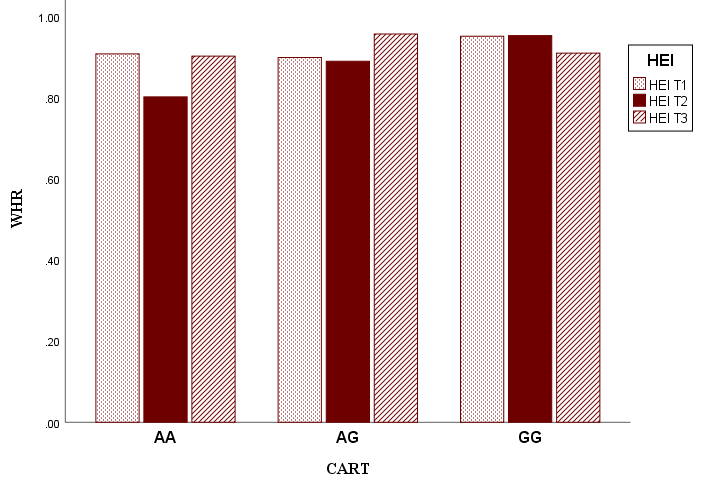

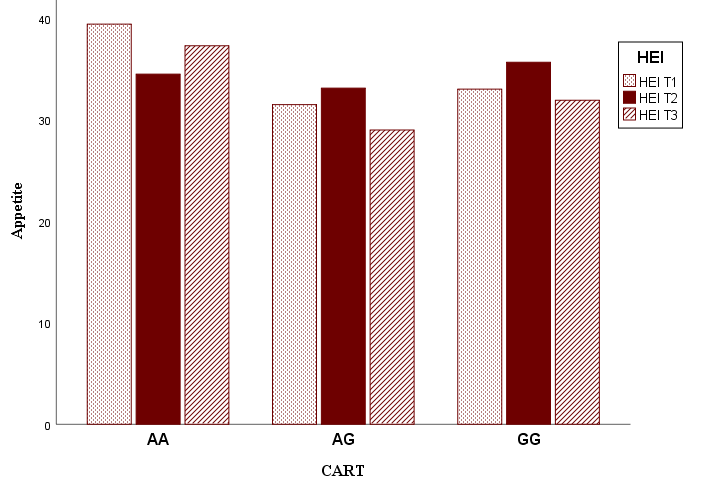

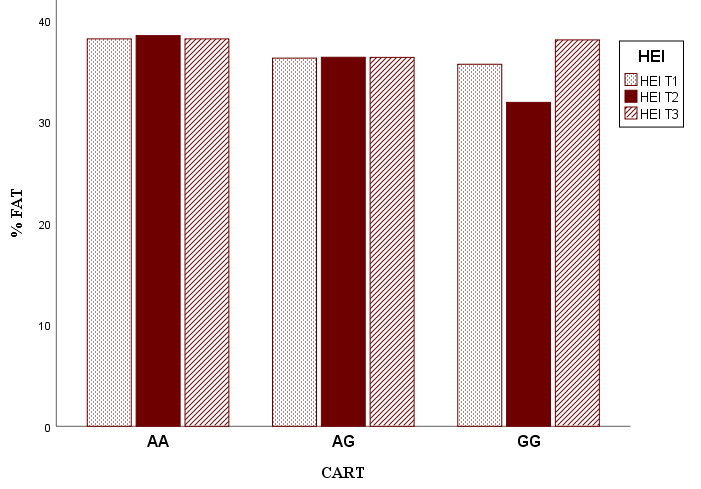

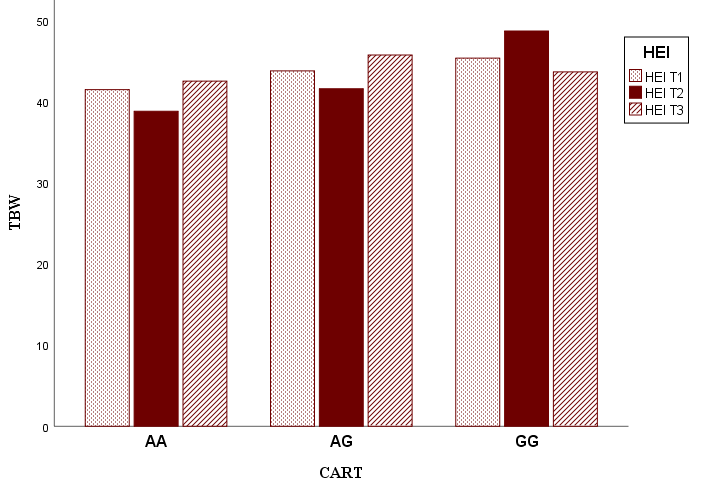


**P _interaction_ = 0.993**

**P _interaction_ = 0.700**

**P _interaction_ = 1.00**

**P _interaction_ = 0.175**

**P _interaction_ = 0.553**

**P _interaction_ = 0.682**

**P _interaction_ = 0.933**

**P _interaction_ = 0.702**

**Figure S1.** P for interaction between HEI, anthropometric and biochemical variables according to CARTPT genotype.


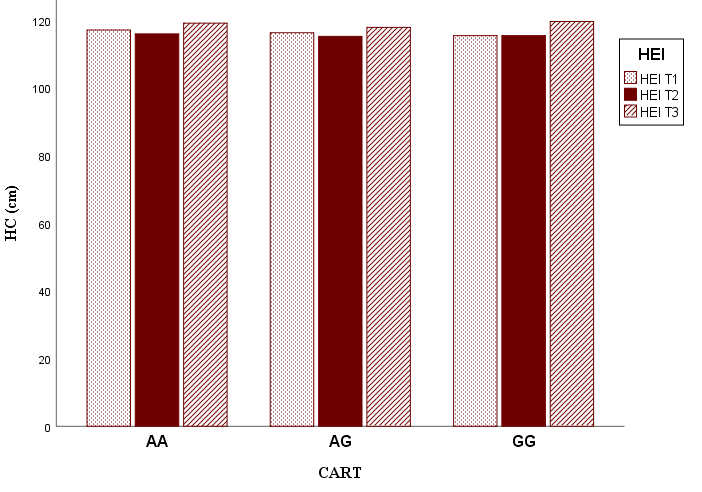

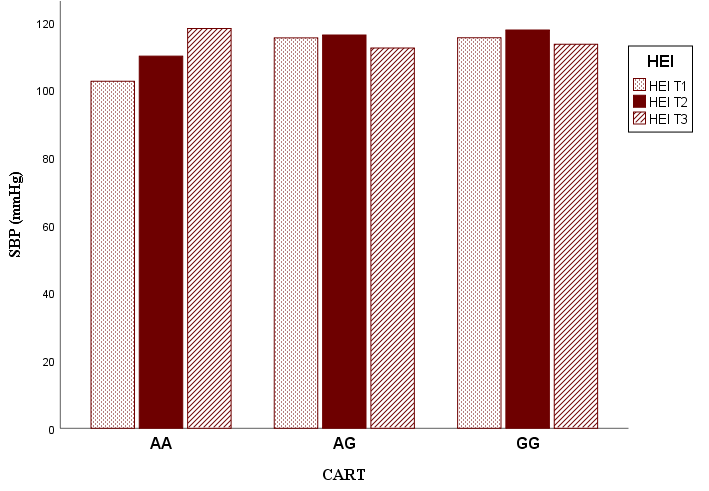

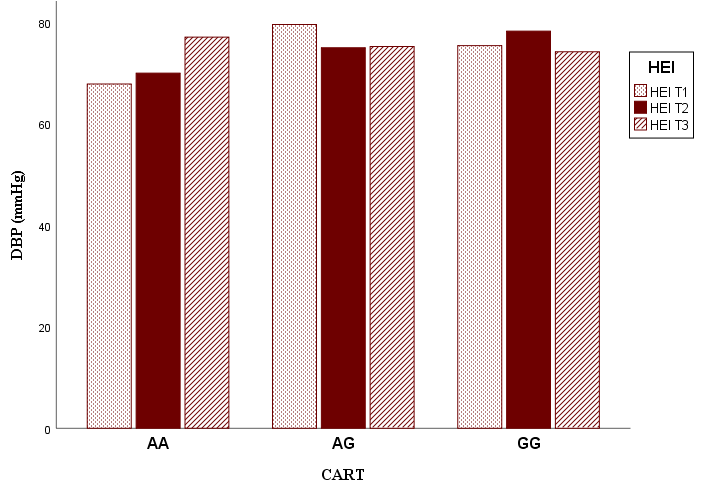

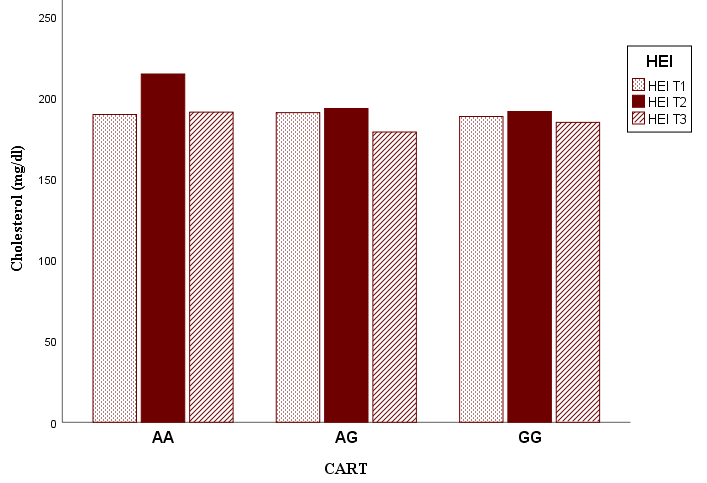

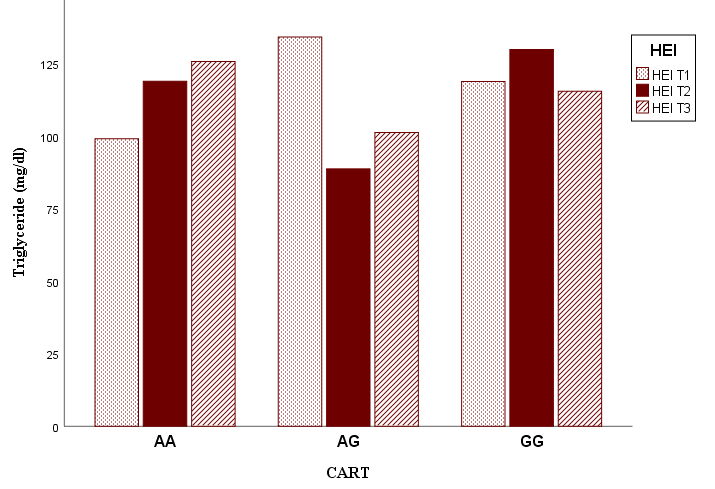

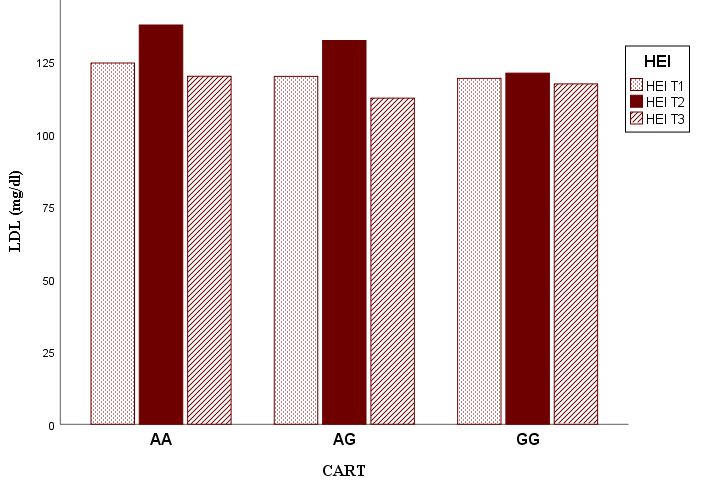

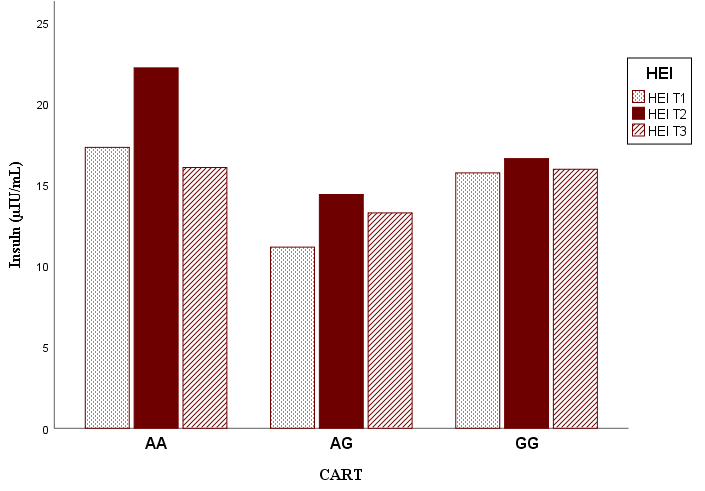

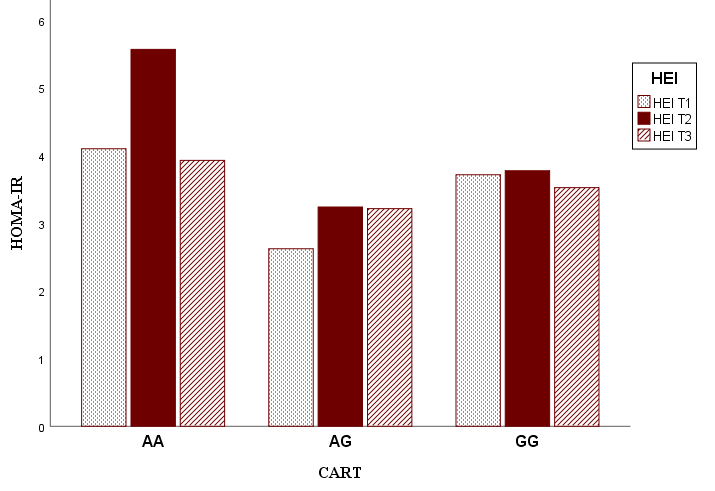


**P _interaction_ = 0.711**

**P _interaction_ = 0.658**

**P _interaction_ = 0.650**

**P _interaction_ = 0.921**

**P _interaction_ = 0.857**

**P _interaction_ = 0.490**

**P _interaction_ = 0.906**

**P _interaction_ = 0.760**

**Figure S1-cont’d.**


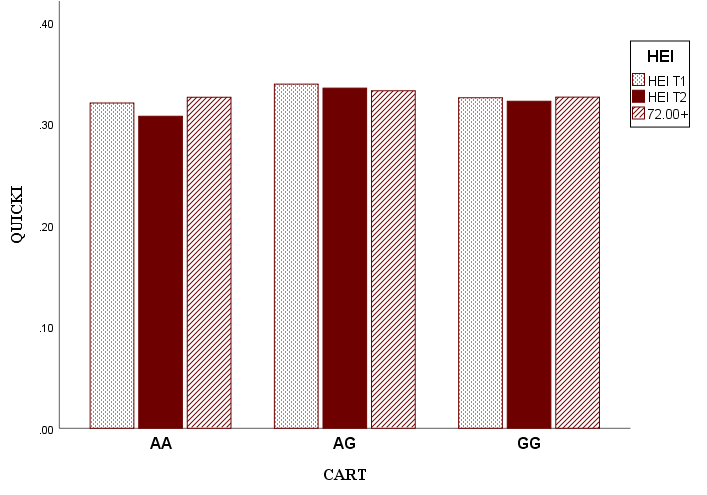

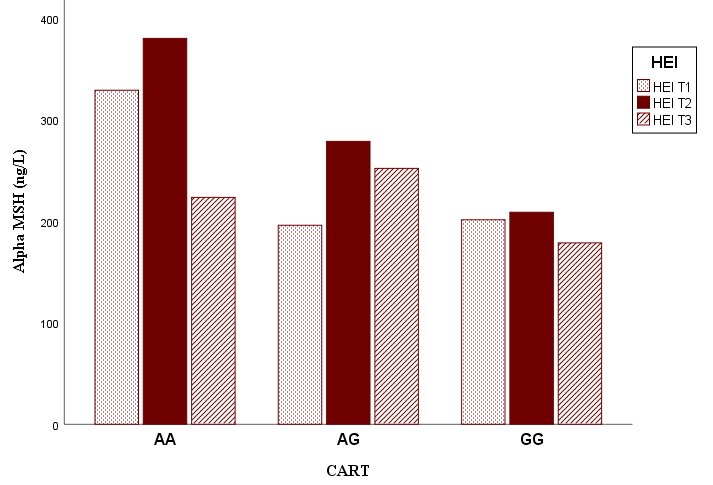

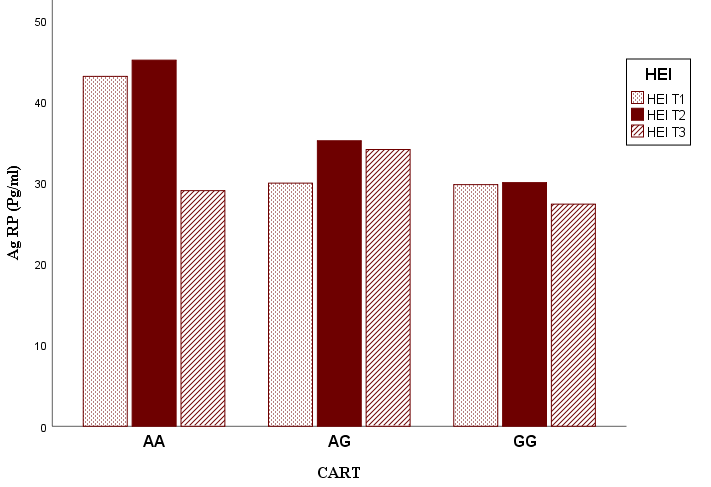


**P _interaction_ = 0.270**

**P _interaction_ = 0.310**

**P _interaction_ = 0.723**

**Figure S1-cont’d.**


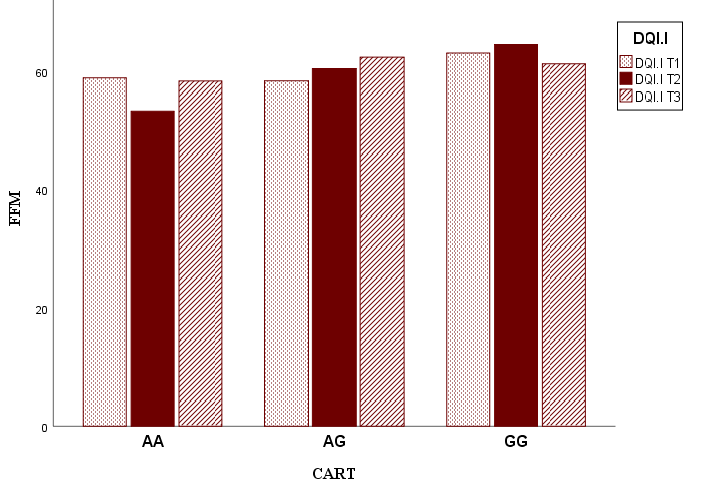

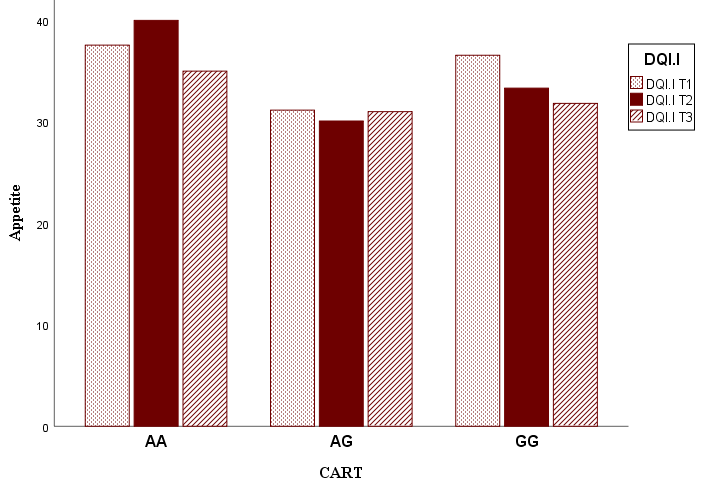

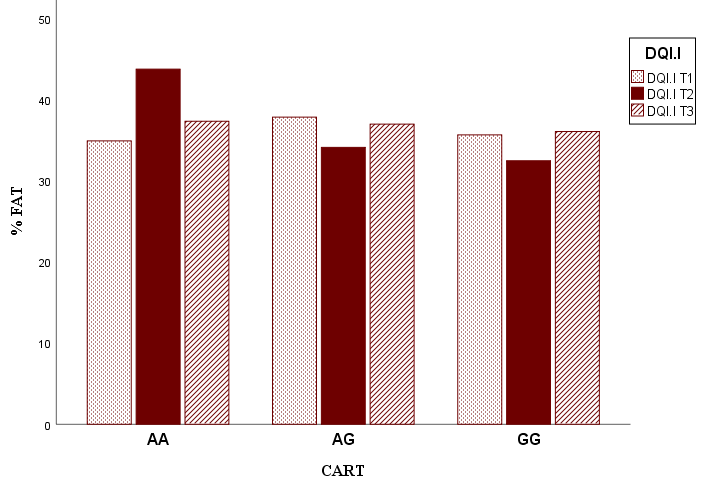

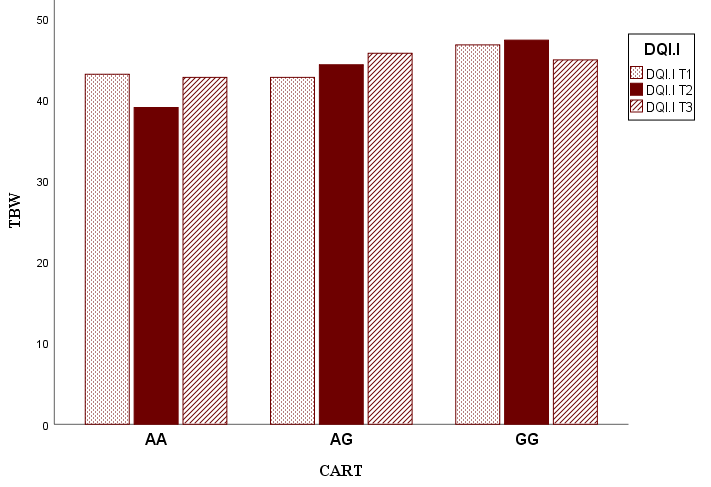

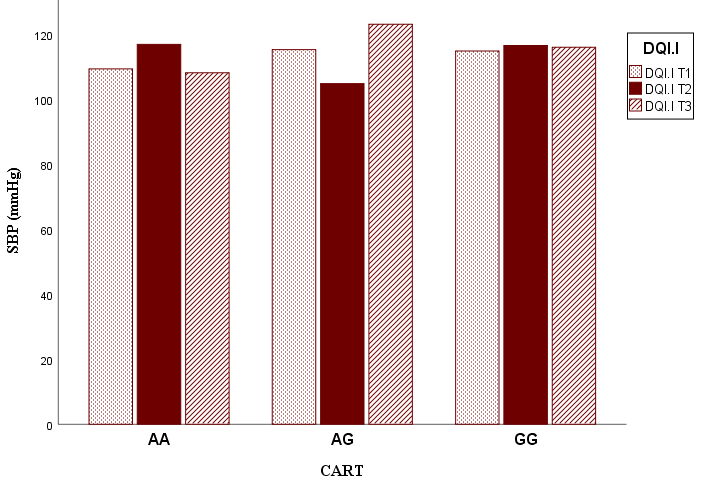

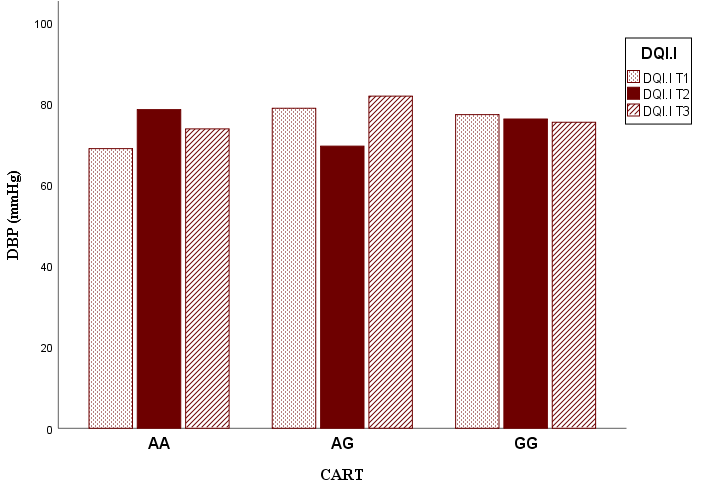

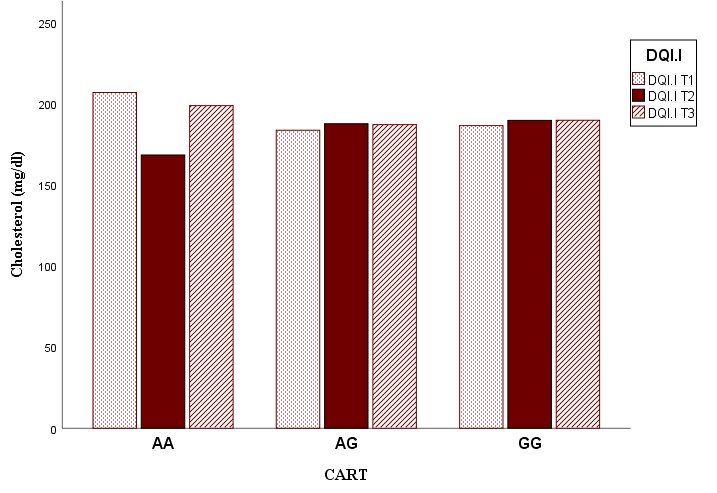

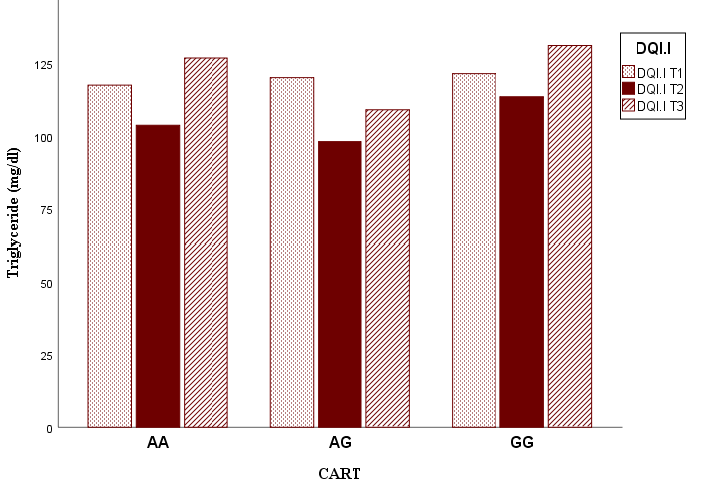


**P_interaction_ = 0.906**

**P_interaction_ = 0.448**

**P_interaction_ = 0.649**

**P_interaction_ = 0.279**

**P_interaction_ = 0.978**

**P_interaction_ = 0.958**

**P_interaction_ = 0.956**

**P_interaction_ = 0.862**

**Figure S2.** P for interaction between DQI-I, anthropometric and biochemical variables according to CARTPT genotype.


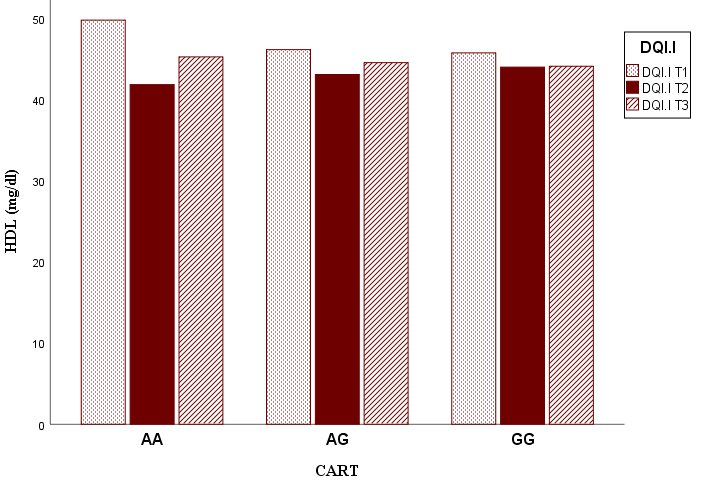

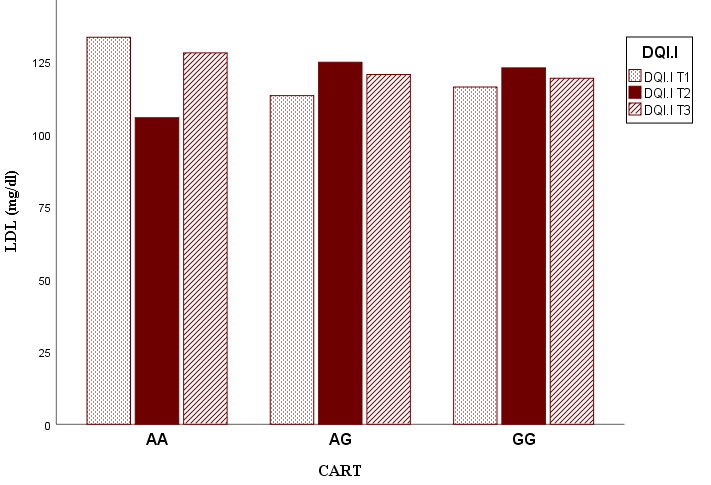

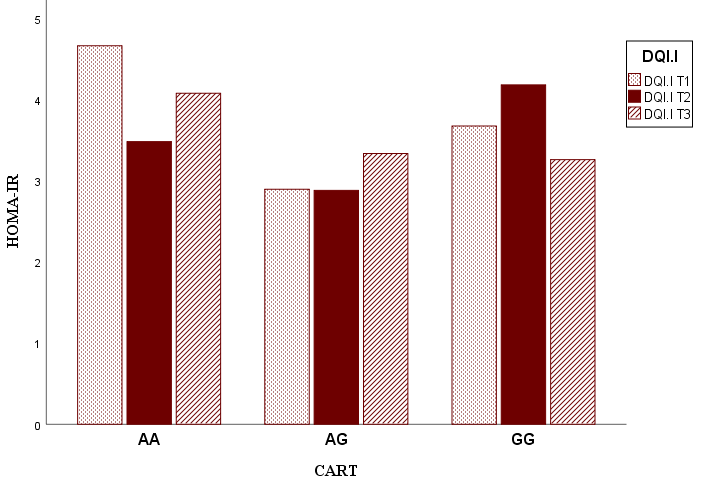

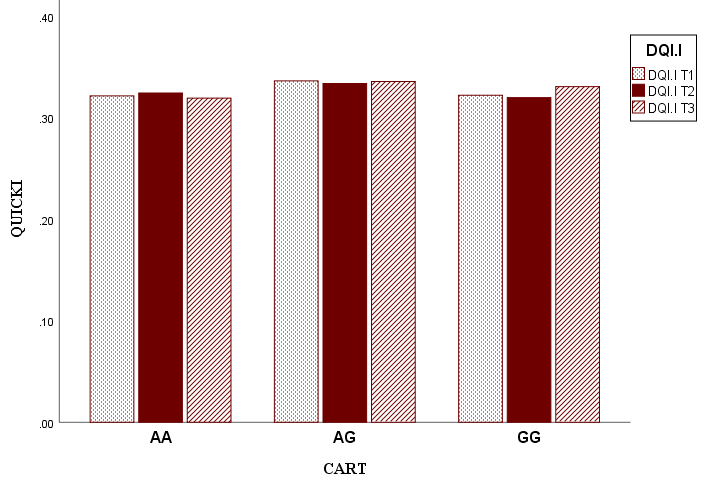

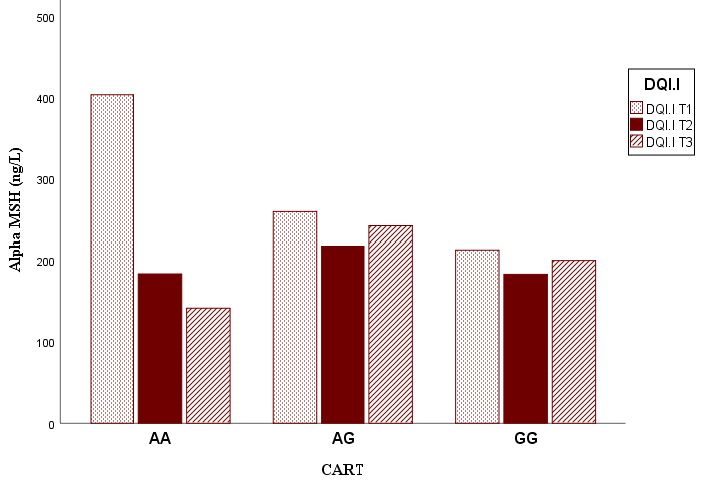

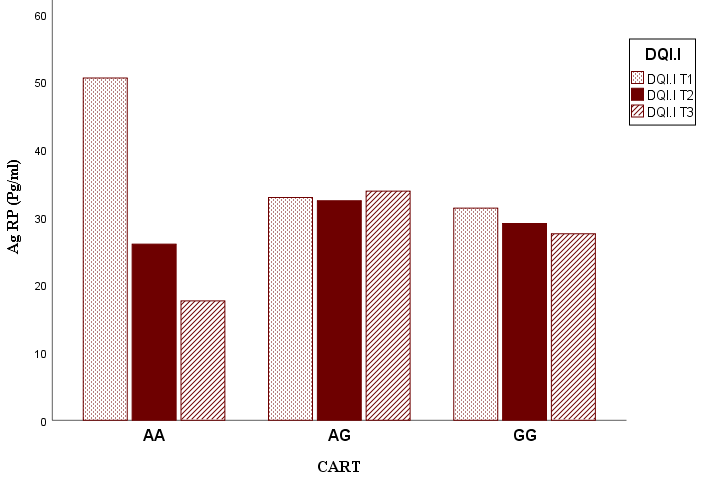

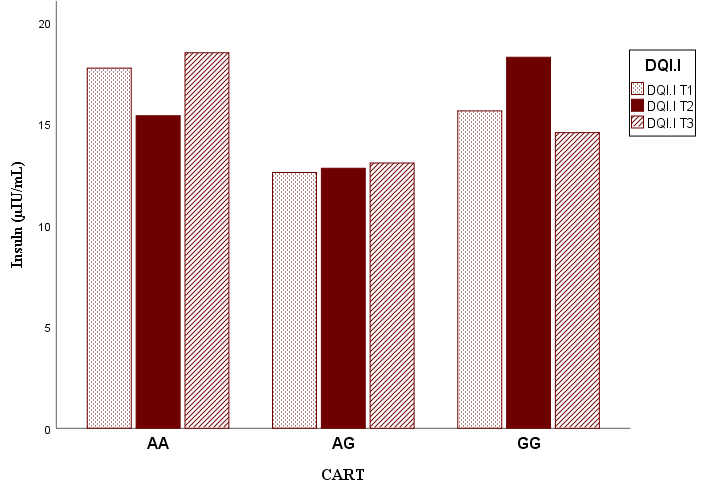


**P_interaction_ = 0.538**

**P_interaction_ = 0.050**

**P_interaction_ = 0.197**

**P_interaction_ = 0.308**

**P_interaction_ = 0.405**

**P_interaction_ = 0.585**

**P_interaction_ = 0.480**

**Figure S2. Cont’d.**
